# Single-cell sequencing and tumorigenesis: improved understanding of tumor evolution and metastasis

**DOI:** 10.1186/s40169-017-0145-6

**Published:** 2017-04-12

**Authors:** Darrell L. Ellsworth, Heather L. Blackburn, Craig D. Shriver, Shahrooz Rabizadeh, Patrick Soon-Shiong, Rachel E. Ellsworth

**Affiliations:** 1 Chan Soon-Shiong Institute of Molecular Medicine at Windber, 620 Seventh Street, Windber, PA 15963 USA; 2 Murtha Cancer Center, Walter Reed National Military Medical Center, 8901 Rockville Pike, Bethesda, MD 20889 USA; 3 NantWorks, 9920 Jefferson Boulevard, Culver City, CA 90230 USA; 4 Murtha Cancer Center, 620 Seventh Street, Windber, PA 15963 USA

**Keywords:** Single-cell sequencing, Whole-genome amplification, Cancer, Tumor heterogeneity, Cancer stem cells, Circulating tumor cells

## Abstract

Extensive genomic and transcriptomic heterogeneity in human cancer often negatively impacts treatment efficacy and survival, thus posing a significant ongoing challenge for modern treatment regimens. State-of-the-art DNA- and RNA-sequencing methods now provide high-resolution genomic and gene expression portraits of individual cells, facilitating the study of complex molecular heterogeneity in cancer. Important developments in single-cell sequencing (SCS) technologies over the past 5 years provide numerous advantages over traditional sequencing methods for understanding the complexity of carcinogenesis, but significant hurdles must be overcome before SCS can be clinically useful. In this review, we: (1) highlight current methodologies and recent technological advances for isolating single cells, single-cell whole-genome and whole-transcriptome amplification using minute amounts of nucleic acids, and SCS, (2) summarize research investigating molecular heterogeneity at the genomic and transcriptomic levels and how this heterogeneity affects clonal evolution and metastasis, and (3) discuss the promise for integrating SCS in the clinical care arena for improved patient care.

## Introduction

The human body is composed of an estimated forty trillion cells [1]. Cellular diversity is controlled by specific RNAs and proteins, whose expression is influenced by exogenous and endogenous signals. While DNA was traditionally thought to be stable, with individual genomes set at the time of fertilization, recent evidence demonstrates that humans are genomic mosaics, comprised of cells that are genetically distinct even though they were derived from a single zygote [2]. Cancer is one of the most common forms of mosaicism in humans, where genetic changes occur in the cancer genome during tumorigenesis. Genomic heterogeneity in cancer is further complicated by the polyclonal nature of most carcinomas, with populations of tumor cells harboring genetic alterations that differ from the host genome and from other cells within the tumor. Intratumor heterogeneity can affect all stages of cancer care from diagnosis through treatment of metastatic disease. Diagnoses based on a single biopsy will likely underestimate the extent of heterogeneity within the tumor and fail to completely detect all clinically-actionable variants, leading to the emergence of drug-resistant populations of cancer cells. Designing therapeutic regimens based solely on characteristics of the primary tumor often fails to effectively treat metastases, which may be descended from minor sub-clones within the primary tumor and/or have acquired new mutations [3]. Therefore, the ability to optimize patient care will depend on a thorough characterization of genomic and transcriptional heterogeneity in cancer at the single-cell level.

**Fig. 1:**
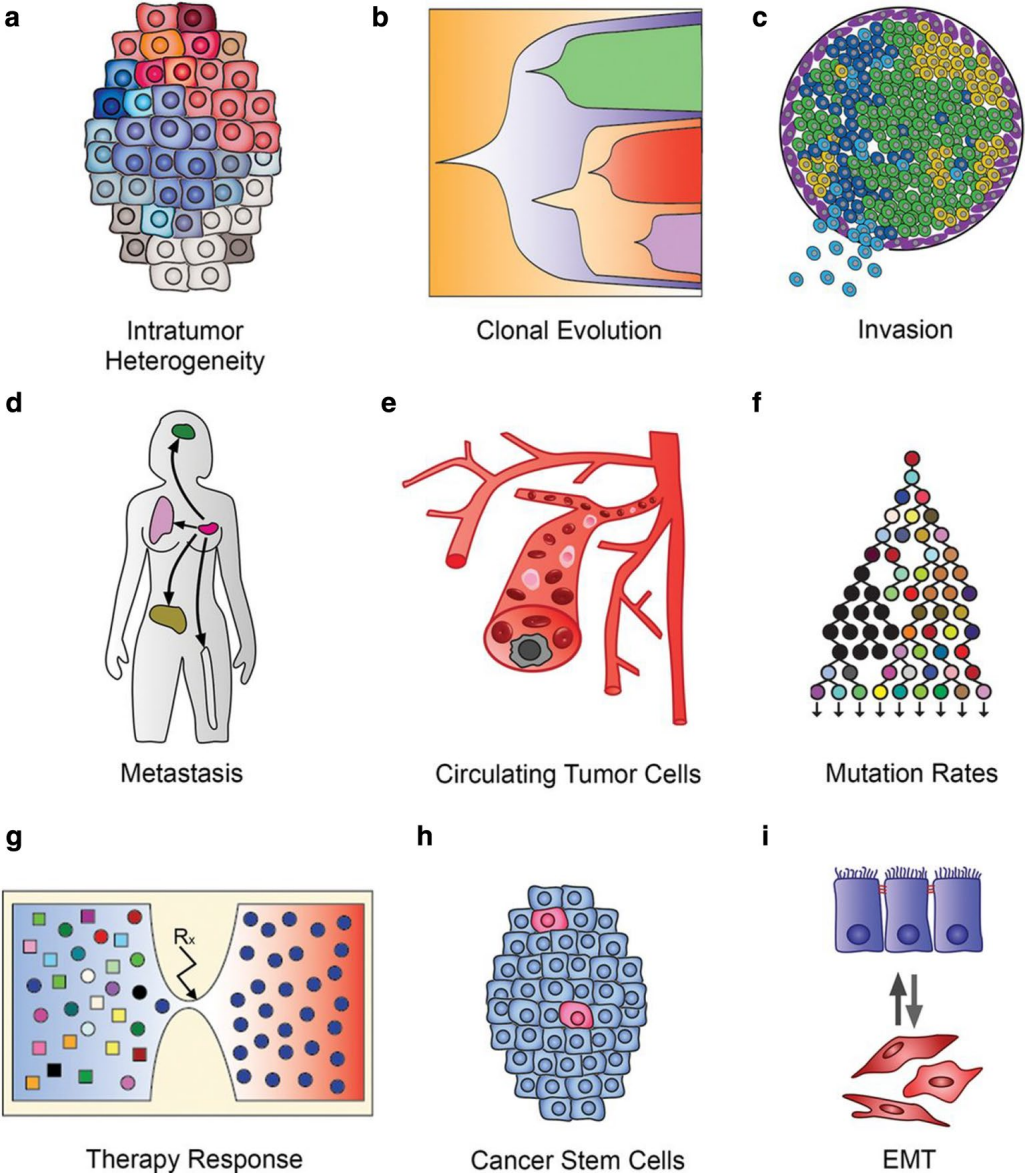
Applications of single-cell sequencing in cancer research. **a** Resolving intratumor heterogeneity; **b** investigating clonal evolution in primary tumors; **c** studying invasion in early stage cancers; **d** tracing metastatic dissemination; **e** genomic profiling of circulating tumor cells; **f** investigating mutation rates and mutator phenotypes; **g** understanding evolution of resistance to therapy; **h** defining cancer stem cells and cell hierarchies; and **i** studying cell plasticity and the epithelial-to-mesenchymal transition [86]

Evaluating genomic heterogeneity at the single-cell level requires overcoming a number of challenges including isolation of individual cells, effective amplification of a single-cell genome to allow for targeted, exome- or genome-wide sequencing, and bioinformatics approaches to discriminate technical artifact from biological differences [4]. The advent of next-generation sequencing (NGS) methods enables researchers to generate genomic, transcriptomic and/or epigenetic data from a single cell (Fig. 1). In this review, we describe (1) current single-cell sequencing (SCS) methodologies and their applications for investigating the important role of genomic and transcriptomic heterogeneity in cancer and (2) how SCS approaches may be incorporated into the clinical arena for improved patient care.

## Single-cell sequencing technologies

SCS is a relatively new technology. The first single-cell RNA sequencing (RNA-seq) data, generated from a single mouse blastomere, were published in 2009 [5], and the first protocol to sequence DNA from single cells was published in 2011 [6]. Generation of whole-genome sequence (WGS), whole-exome sequence (WES), or RNA-seq from single cells requires isolation of individual viable cells or intact nuclei, amplification of minute amounts of DNA or RNA from the cell, sequencing, and analysis of the ensuing data. Continuous advancements in technology over the past 5 years have led to significant improvements in genome coverage and sequence quality, as well as drastic reductions in overall costs.

### Isolation of single cells

**Fig. 2:**
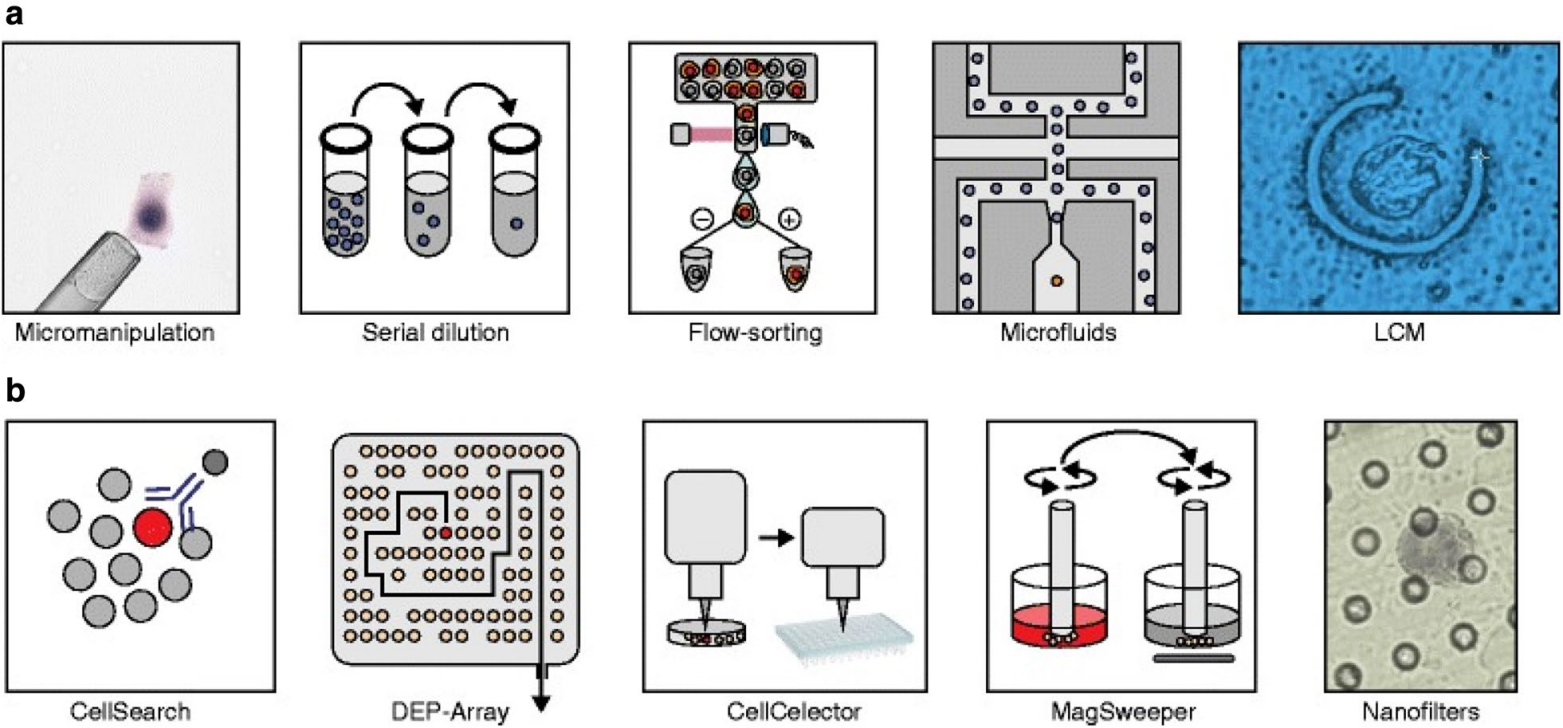
Single-cell isolation methods. **a** Methods for isolating single cells from abundant cell populations include: robotic or manual micromanipulation, serial dilution, flow-sorting, microfluidic methods, and laser-capture microdissection; **b** methods for isolating single cells from rare cell populations include: CellSearch™, DEP-Array™, CellCelector™, MagSweeper™, and nanofilters [16]

A summary of methods for isolating single cells is presented in Fig. 2. Serial dilution provides a simple, low-cost method for isolating individual cells from abundant cell populations but is time consuming and requires expertise [7]. Micromanipulation and laser capture microdissection (LCM) both rely on visualization of the cells using a microscope. While LCM has the advantage of preserving spatial relationships within a tissue specimen, the tissue must be sectioned, often at thicknesses smaller than the diameter of single cell, leading to loss of chromosomal material [8]. Flow-assisted cell sorting and microfluidic platforms represent high throughput approaches that utilize specific properties of the cells, such as size or expression of biomarkers, for isolating individual cells from cellular suspensions of fresh tissue [9]. The approaches outlined above are sufficient for isolating single cells from tissue sections or large populations of cells in culture, but are not effective for isolating rare cells such as circulating tumor cells (CTCs) in peripheral blood or disseminated tumor cells (DTCs) in bone marrow.

In contrast to the relatively non-specific methods mentioned above, numerous techniques have been developed for targeting and isolating single rare cancer cells from large populations of histologically diverse cells such as peripheral blood (Fig. 2). The CellSearch™ system is the only FDA-approved cell isolation and enumeration system currently available. An important component of the system is the CellSearch^®^ Epithelial Cell Kit, which contains magnetic capture particles with a surface layer coated with antibodies targeting epithelial markers including leukocyte common antigen (CD45−), epithelial cell adhesion molecule (EpCAM+), and cytokeratins 8, 18+, and 19+. Rare CTCs are isolated from whole blood and enriched by exposing the buffy layer to the capture particles. During incubation, CTCs bind to the capture particles, are magnetically separated from unbound cells, and are then enumerated by fluorescence staining [10].

MagSweeper™ is an automated system that also uses immunomagnetic separation to purify rare cells in circulation. A magnetic rod is robotically swept through a sample containing labeled cells from peripheral blood to specifically capture circulating epithelial cells. Sequential rounds of cell capture-wash-release-recapture result in an enrichment of epithelial cells by 10^8^-fold. Purified cells can be individually selected for subsequent biochemical analysis [11].

The DEP-Array™ system combines size and cell-surface expression properties for cell isolation. DEP-Array™ achieves CTC enrichment by density gradient centrifugation followed by staining with antibodies directed against CD45− and various cytokeratins. CTCs with the appropriate epithelial cell morphology and staining patterns are then recovered for molecular assessment [12].

CellCelector™ is a technique that uses automated micromanipulation for isolating individual cells from dense, single-cell microarrays. A suspension of cells from culture (or peripheral blood) is deposited on custom-made arrays containing micro-wells, controlling distribution and density to deposit one cell in each well. The array is then screened by a process known as micro-engraving—the array is covered with a glass slide coated with mono-clonal antibodies (goat anti-mouse IgA and IgG) and incubated. Using a microarray scanner, the glass slide can be interrogated for antibodies of interest that were secreted by the cells in the corresponding wells. Areas on the glass slide serve as a guide to locate matching micro-wells and individual cells in the wells can be selected by micromanipulation for subsequent analysis [13].

Because some of these isolation methods rely on cell-surface markers such as EpCAM and other epithelial proteins, these systems may not detect all rare cancer cells, including those that have undergone epithelial-to-mesenchymal transition (EMT). The CellSieve™ technique uses size discrimination to separate and isolate cells, and thus may be useful for capturing CTCs that are frequently larger than white blood cells [14].

### Whole-genome amplification

The minute amount of DNA (~6 pg) and RNA (~10 pg) isolated from a single diploid cell requires whole-genome amplification (WGA) or whole-transcriptome amplification (WTA) to generate sufficient material for NGS. In recent years, numerous methods have been developed to amplify the DNA or RNA in a single cell with a focus on minimizing technical artifacts, such as preferential amplification of certain regions and/or allelic loss, and providing complete coverage of the genome [8, 15–17].

**Fig. 3:**
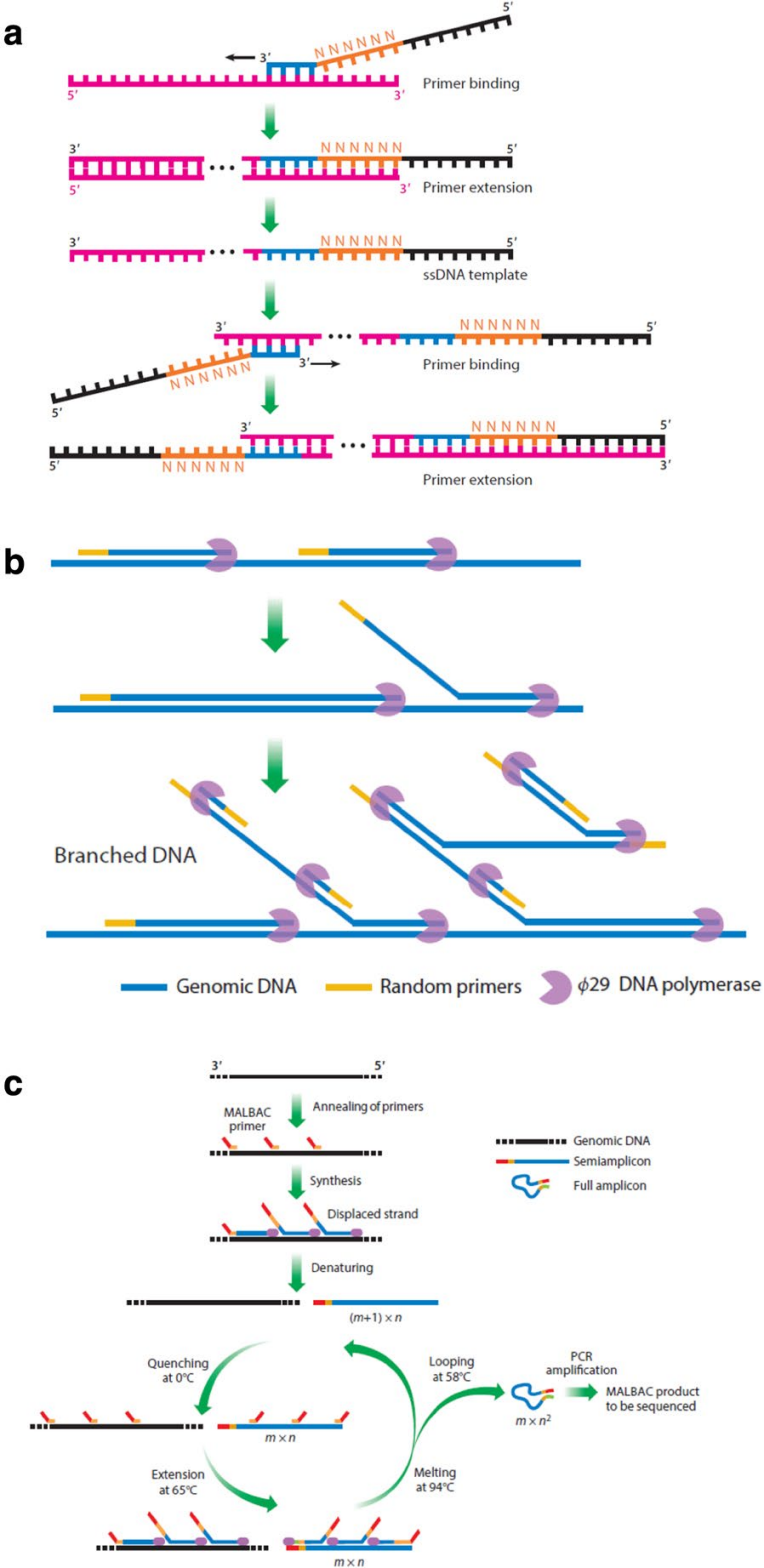
Main approaches used for whole-genome amplification of single cells. **a** Degenerate Oligonucleotide-primed polymerase chain reaction (DOP-PCR) uses primers with common sequences at the 5′- and 3′-ends, but six random nucleotides near the 3′-end to allow hybridization at many sites throughout the genome; **b** multiple displacement amplification (MDA) uses φ29 DNA polymerase and random primers in a non-PCR based amplification reaction in which newly-synthesized strands are displaced from the original DNA molecule and serve as templates for additional DNA synthesis, resulting in a hyper-branched network; **c** multiple annealing and looping based amplification cycles (MALBAC) uses random primers with a common sequence at the 5′-end to amplify only the original template DNA and semi-amplicons. Full amplicons have complementary ends that allow the formation of closed-loop structures that prevent further amplification [15]

Currently, three main approaches are used for WGA (Table 1). In the degenerate oligonucleotide-primed polymerase chain reaction (DOP-PCR) method, amplification is initiated with primers that share defined sequences at the 5′- and 3′-ends but contain six variable nucleotides (all possible combinations of A, C, G, and T) near the 3′-end to allow dense, even hybridization to the template DNA [18]. During the initial five to eight cycles of amplification, the defined and variable nucleotides at the 3′-end of the primers bind to the DNA template at many sites throughout the genome, followed by strand extension. In the second stage of amplification, the previously generated amplicons are amplified using primers that target the common sequence at the 5′-end of the primers [15] (Fig. 3a). High amplification bias, in which only certain regions of the genome are preferentially amplified and thus amenable to large-scale sequencing, results in relatively low coverage of the genome (~10%), making DOP-PCR useful for copy-number assessment in single cells but undesirable for single nucleotide variant (SNV) detection [16].

**Table 1:** Comparison of whole-genome amplification methods for single-cell DNA sequencing. Adapted from Liang et al. [8]

Method	Enzyme used	Application	Genome coverage	SNV detection	CNV detection	Amplification bias
DOP-PCR	*Taq* DNA polymerase	Single nucleus sequencing	Low (~10%)	High false negative and false positive rates	Useful	High (10^2^–10^6^ fold)
MDA	φ29 DNA polymerase; *Bst* DNA polymerase	Single nucleus exome sequencing	Moderate (>70%)	Useful but has a high false negative rate due to amplification bias	Not accurate	Moderate (3- to 4-fold)
MALBAC	*Bst* DNA polymerase	Single-cell genome/exome sequencing	High (>90%)	High false positive rate due to low fidelity	Accurate	Low

*SNV* single nucleotide variant, *CNV* copy number variant, *DOP*-*PCR* degenerate oligonucleotide-primed polymerase chain reaction, *MDA* multiple-displacement amplification, *MALBAC* multiple annealing and looping based amplification cycles

Multiple-displacement amplification (MDA) is a non-PCR based amplification technique that does not require thermal cycling, in which random hexamer primers are annealed to denatured DNA from a single cell to synthesize new DNA strands [19]. As the polymerase advances, newly-synthesized strands are displaced from the original DNA molecule and serve as templates for further primer annealing and additional DNA synthesis, resulting in a hyper-branched network and exponential amplification (Fig. 3b). DNA synthesis is normally catalyzed by φ29 DNA polymerase, an isothermal enzyme capable of generating quality DNA with high coverage of the genome for use in SCS. MDA works best for mutation detection but is not sufficient for copy number analysis due to moderate amplification bias and non-uniform genome coverage.

The multiple annealing and looping based amplification cycles (MALBAC) method utilizes a quasi-linear pre-amplification step to decrease amplification bias [20]. An important strategy of the MALBAC method involves amplification using only the original template DNA, rather than exponential amplification, by protecting the amplification products (Fig. 3c). Amplification using *Bst* (*Bacillus stearothermophilus*) polymerase is initiated with primers that share a common 27-nucleotide sequence at the 5′-end but contain eight variable nucleotides at the 3′-end to allow random hybridization to the template DNA. A polymerase with strand displacement activity first synthesizes semi-amplicons of variable length, which dissociate from the template at high temperature. Amplification of the semi-amplicons generates full amplicons with complementary ends that allow the formation of closed-loop structures, which prevent the full amplicons from being used as template. The full amplicons can then be exponentially amplified by PCR to generate microgram quantities of DNA for NGS. MALBAC provides high uniformity in coverage across the genome (93% coverage of at least 1X at a mean sequencing depth of 25× for a single human cell) and is useful for detecting copy number variants (CNVs) in single cells; however, MALBAC has a high false positive error rate and is not appropriate for detecting point mutations [8].

### Whole-transcriptome amplification

**Fig. 4:**
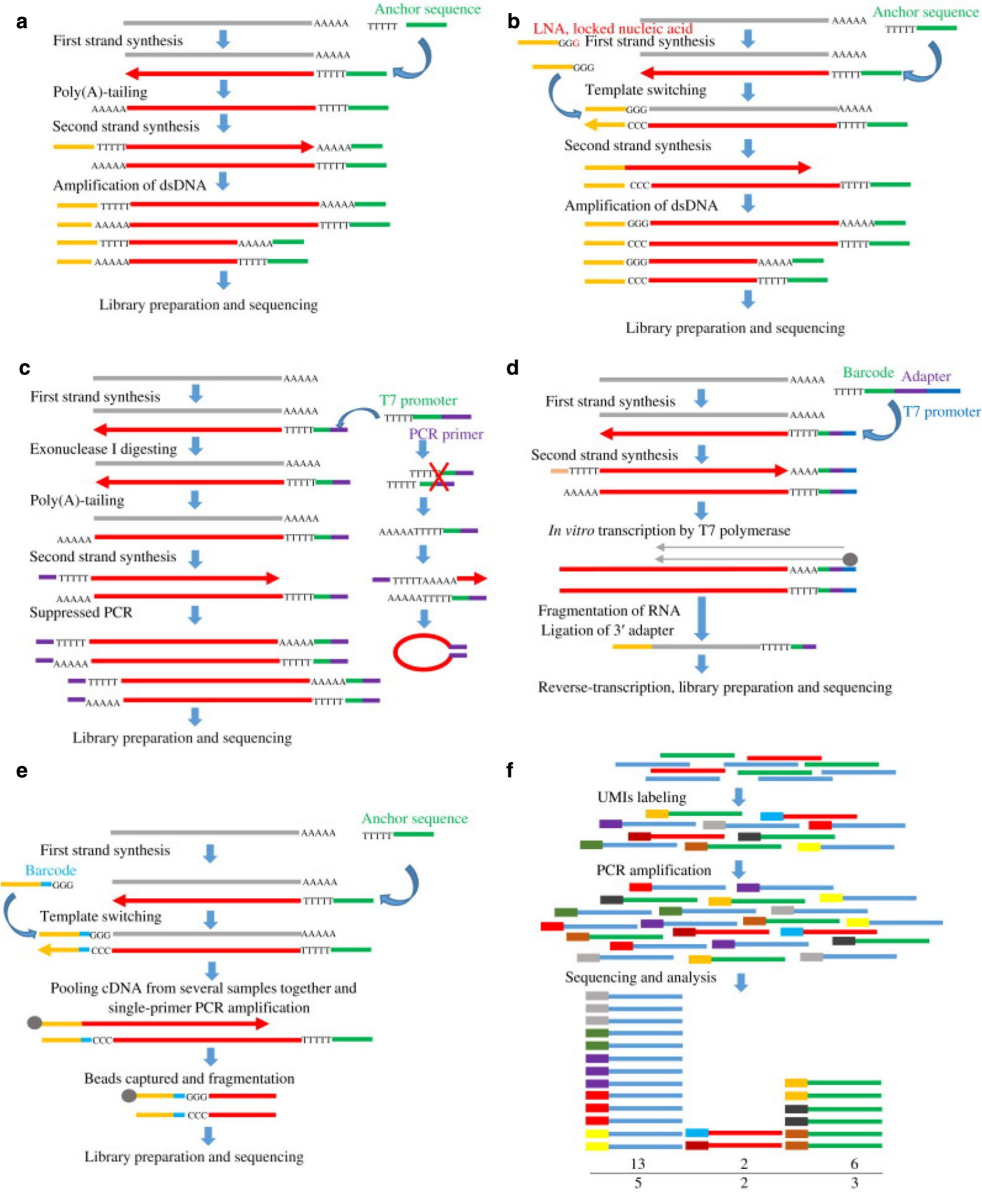
Main approaches used for whole-transcriptome amplification of single cells. **a** The Tang method performs reverse transcription of mRNA for single-cell RNA-seq using an oligo-dT primer with an anchor sequence, then a poly-A tail is added to the 3′-end of the first cDNA and the second strand is synthesized using a different oligo-dT primer with a different anchor sequence; **b** Smart-seq and Smart-seq2 implement a template-switching step to increase the number of full-length cDNA transcripts with an intact 5′-end; **c** quartz-seq limits amplification of unwanted byproducts by removing excess primer with exonuclease I before second-strand synthesis and using suppression PCR to form hairpin structures that cannot be amplified; **d** cell expression by linear amplification and sequencing (CEL-Seq) includes a template-switching step and uses molecular barcodes and pooling of samples from multiple single cells prior to linear amplification; **e** single-cell tagged reverse transcription (STRT) permits multiplex sequencing of multiple cells in the same reaction using a template-switching mechanism to simultaneously introduce a molecular barcode and an upstream primer-binding sequence during reverse transcription; **f** quantitative single-cell RNA-seq generates full-length transcripts using template switching and incorporating random UMI (unique molecular identifier) sequences to label individual cDNA molecules and eliminate amplification bias [8]

A number of approaches have been developed for WTA of single cells (Fig. 4; Table 2; reviewed in [8]). The basic steps include reverse transcription of messenger RNA (mRNA) to complimentary DNA (cDNA) followed by cDNA amplification via PCR [9]. Tang and colleagues [5] first described a method for single-cell RNA-seq in which reverse transcription was performed using an oligo-dT primer with an anchor sequence, then a poly-A tail was added to the 3′-end of the first cDNA. The second strand was synthesized using a different oligo-dT primer with a different anchor sequence, and the cDNA was amplified by PCR.

**Table 2:** Comparison of single-cell transcriptome sequencing methods. Adapted from Liang et al. [8] and Navin [16]

Method	Reverse-transcription enzyme used	WTA method	Reverse-transcript size	Position bias
Tang’s method	Reverse transcriptase	Poly-A tailing	0.5–3.0 kb	3′-end
Smart-seq/Smart-seq2	M-MLV RT	Template-switching; locked nucleic acid in Smart-seq2	Full-length	Low 3′-end
Quartz-seq	Reverse transcriptase	Poly-A tailing; suppression PCR	0.4–4.0 kb	3′-end
CEL-seq/CEL-seq2	In vitro transcription	Poly-A tailing; barcoding	3′-end only	High 3′-end
STRT	Reverse transcriptase	Template-switching; barcoding	Full-length, only detect 5′-end	5′-end

*WTA* whole-transcriptome amplification, *SMART* switching mechanism at the 5′-end of RNA template, *M*-*MLV RT* Moloney murine leukemia virus reverse transcriptase, *CEL*-*Seq* cell expression by linear amplification and sequencing, *STRT* single-cell tagged reverse transcription sequencing

Smart-seq and Smart-seq2 (switching mechanism at the 5′-end of the RNA transcript) represent variations of this approach designed to reduce 3′-bias, increase cDNA yields and the number of full-length transcripts, and detect alternative splice sites, novel exons, and genetic variants [21, 22]. These techniques implement a template-switching step, which increases the number of transcripts with an intact 5′-end. During first-strand synthesis, the reverse-transcriptase enzyme, isolated from the Moloney murine leukemia virus, adds extra cytosine (C) nucleotides to the 5′-end of the cDNA. By adding a primer containing guanine (G) nucleotides, the enzyme will switch templates and reverse-transcribe to the end of the primer, resulting in a full-length cDNA molecule that contains the complete 5′-end of the mRNA and an anchor sequence that will serve as a universal priming site for second-strand synthesis. Smart-seq2 contains technological improvements to increase sensitivity, accuracy, and the number of full-length transcripts.

Quartz-seq was developed to improve reproducibility and sensitivity of SCS methods to quantify the heterogeneity of gene expression between cells. Quartz-seq focuses on limiting the amplification of unwanted byproducts by removing excess primer with exonuclease I before second-strand synthesis, restricting poly-A tailing, and using suppression PCR, which permits short DNA fragments to form a hairpin structure that cannot be amplified [23]. Similar to other poly-A tailing methods for WTA of single cells, Quartz-seq shows a weak 3′-bias but is capable of detecting differentially expressed genes between different cell types.

The cell expression by linear amplification and sequencing (CEL-Seq) method overcomes challenges posed by the minute amount of RNA in a single cell by including a template-switching step and using molecular barcoding (attaching a short unique sequence to template DNA or RNA molecules to uniquely identify each molecule) and pooling of samples prior to linear amplification of mRNA in one round of in vitro transcription [24]. Subsequent modifications (CEL-Seq2), including shortening the CEL-Seq primer, optimizing the conversion of RNA to dsDNA, and ligation-free library preparation, have increased the efficiency, sensitivity, and cost-effectiveness of the method [25]. Despite recent improvements, these approaches still suffer from 3′-amplification bias, and therefore may not detect variable transcripts.

Unlike other whole-transcriptome amplification methods, single-cell tagged reverse transcription (STRT) is a highly multiplexed method for single-cell RNA-seq that quantifies gene expression in single cells by sequencing the 5′-ends of mRNA. STRT uses a template-switching mechanism to simultaneously introduce a molecular barcode and an upstream primer-binding sequence during reverse transcription, which permits multiplex sequencing of multiple cells simultaneously. STRT provides the ability to identify the transcription start site, locate promotor and enhancer elements, and conduct large-scale quantitative analysis but is not suitable for detecting alternatively-spliced transcripts [26].

### Sequencing considerations

Despite recent progress, SCS techniques currently being used in research have technological limitations. Amplified DNA from single cells may be subjected to targeted sequencing, WES, or WGS. Targeted sequencing is associated with a lower false positive rate, with more uniform coverage of the targeted areas. In contrast, WES and WGS provide greater coverage of the genome and an increased ability to discover mutations; however, as genome coverage increases so does the false positive rate. WGS of single cells provides the greatest opportunity to detect genetic alterations across the genome but at significantly increased cost [4].

### Data analysis

Single-cell isolation techniques and WGA/WTA may introduce artifacts that must be considered when analyzing sequencing data. Based on the cell selection approach utilized, cells may be biased in size, rates of cell division, or cellular properties. WGA techniques result in low physical coverage of the genome, allelic dropout (where one or both alleles at a heterozygous locus fail to amplify and therefore are not detected), uneven genome coverage, and false-positive and false-negative errors. For RNA-seq, reverse transcription of mRNA to cDNA followed by cDNA amplification via PCR introduces technical artifact and amplification bias, particularly for lower-abundance transcripts. In fact, only ~10–20% of transcripts are reverse transcribed with current methods and many transcripts are not full-length [9]. Comparing SCS results to bulk tumor sequence can be used to estimate technical errors; however, this approach may decrease the ability to detect variants specific to the single cells. Incorporating molecular barcodes, also known as unique molecular indices or UMIs, may prove useful for improving efficiency and distinguishing true mutations from PCR or sequencing errors [27]. New algorithms and computational methods to address these limitations are currently being developed and may provide the necessary informatics infrastructure to accurately and reliably analyze SCS data.

## Single-cell sequencing of tumor cells

### Cancer stem cells

Normal stem cells are rare, quiescent cells that survive in an undifferentiated state for extended periods of time and have the capacity for unlimited self-renewal and the ability to generate morphologically diverse progeny cells [28]. Tissue-specific stem cells that reside in differentiated tissues are important in growth and development because they also have the capacity for self-renewal and the ability to differentiate into a variety of specific cell types. Tissue-specific stem cells may accumulate certain mutations over time that initiate carcinogenesis, causing them to become cancer stem cells. Additional mutations in cancer stem cells that alter molecular pathways influencing genome stability, resistance to apoptosis, and normal growth and differentiation, may occur during tumorigenesis, leading to substantial genetic and functional diversity among clonal populations of cells within a primary carcinoma [29, 30]. Although the development of genetic diversity in cancer stem cells has not been well defined, SCS is now being used to study cancer stem cells to identify mutations in key functional pathways promoting tumorigenesis [31]. Because cancer stem cells are believed to be responsible for many aspects of cancer biology such as tumorigenesis, metastasis, and drug resistance, eradication of these stem cells has become a prime objective of modern anti-cancer therapeutics.

The ability to quantify cell-to-cell variation in gene expression using single-cell RNA-seq is important to understanding clinical parameters such as a patient’s response to treatment and the potential for disease recurrence. As a result, research on cancer stem cells at the individual cell level has accelerated in recent years, focusing on unique functional properties, including extensive cell-to-cell heterogeneity in gene expression and plasticity in the degree of “stemness” [32]. Single-cell transcriptome analysis of cancer stem cells has been difficult due to their rarity and the small amount of total RNA in a single cell; however, recent developments in single-cell isolation, WGA, and RNA-seq discussed above [33] provide an opportunity to study the transcriptomes of these rare stem cells and provide insight into the complex nature of functional heterogeneity at the individual cell level [34].

In breast cancer, single-cell gene expression profiling has been used to identify regulatory networks influencing differentiation, stemness, pluripotency, EMT, and proliferation, which are important for the identification of rare cell types such as stem cells [35]. Investigating the potential role of stem cells in the initiation and progression of breast cancer metastases, Lawson and colleagues [36] developed a fluorescence-activated cell sorting assay to identify human metastatic cells from a patient-derived xenograft (PDX) mouse model. Multiplex analysis detected heterogeneity in gene expression and revealed a distinct stem-cell-like gene expression signature in early stage metastatic breast cancer cells, suggesting that breast cancer metastases may be initiated by stem-like cells. Paired-end transcriptome sequencing identified unique patterns of gene expression in breast cancer stem cells compared to other breast cancer cell types that may regulate the effects of oncogenes and tumor suppressor genes [37].

Using single-cell RNA-seq to profile 430 cells from five primary glioblastomas, Patel et al. [38] found variability among cells in patterns of gene expression in pathways such as oncogenic signaling, proliferation, and immune response. Importantly, an examination of “stemness” genes identified a continuous, rather than discrete, stemness-related gene expression signature among individual glioblastoma cells, which suggests that glioblastomas contain primitive populations of stem-like cells with variable degrees of differentiation and proliferative capacity.

### Primary tumors

#### Breast cancer

A summary of SCS studies on primary tumors from a variety of human cancers is presented in Table 3. The first report of SCS in cancer published in 2011 [6] performed copy number evaluation on flow-sorted nuclei from two triple-negative breast carcinomas. One tumor was found to be highly mono-genomic and was composed of cells representing a single clonal expansion, but the other carcinoma was genetically heterogeneous, containing distinct clonal subpopulations of cells that were hypothesized to have originated early in tumor development. Further single-cell studies supported this concept that CNV tends to occur early in the development of breast cancer. Wang and colleagues [39] evaluated nuclei from cells undergoing cell division (G2/M nuclei) to examine clonal diversity and mutational evolution in two breast cancer patients. No two single cells from a luminal A or triple negative breast tumor exhibited identical genomic profiles even though the mutation rate was significantly higher in the triple negative carcinoma (>13-fold). Alterations in copy number were widely shared, suggesting they occurred early in carcinogenesis, while point mutations appeared to evolve gradually over a longer period of time. A follow-up study using single-nucleus sequencing of 1000 single cells from 12 patients with triple-negative breast cancer identified one to three major clonal subpopulations in each tumor that shared a common evolutionary lineage and were unlikely to result from gradual accumulation of CNVs over time [40]. Similarly, in two patients with estrogen receptor (ER)-positive breast cancer, chromosomal alterations characteristic of ER+ tumors including duplications of 1q and 8q and deletion of 11q were shared across most single cells from both patients, indicating that these events occurred early in the development of these tumors [41]. Together, the SCS data suggest that the earliest steps of tumor development involve copy number changes that occur in punctuated bursts, but point mutations evolve gradually, driving clonal expansions and generating extensive clonal diversity within a primary carcinoma.

**Table 3:** Summary of single-cell sequencing studies on primary tumors from a variety of human cancers

Tumor	Tissue source (number of cells, patients/cell lines)	Data type	Results	Reference
*Breast*				
	TNBC (200, 2)	CNV	TNBC displays punctuated clonal evolution where CNVs are shared across single cells	[6]
	TNBC (66, 1), ER + HER2- (113, 1)	CNV and SNV	TNBC has a higher mutation rate than ER + HER2- tumors or normal cells; CNVs are an early event in tumorigenesis	[39]
	TNBC (1000, 12)	CNV	Supports theory of punctuated clonal evolution	[40]
	ER + (332, 2)	CNV	Supports theory of punctuated clonal evolution	[41]
	MDA-MB-231 and CN34 cell lines (44, 2)	RNA-seq	Rare cell populations with highly variable gene expression differences have increased metastatic capacity and ability to survive treatment	[42]
	MDA-MB-231 cell line (15, 1)	RNA-seq	Development of drug-resistance to paclitaxel is associated with unique mutations; gene expression changes not detectable in bulk tumors	[43]
	HER2 + (8, 2)^a^	RNA-seq	404 genes differentially expressed in breast cancer stem cells, including CA12 which may be prognostic	[37]
*Lung*				
	Lung adenocarcinoma PDX (34, 1)	RNA-seq	Gene expression profiling identifies a subpopulation of PDX cells with poor prognosis	[44]
	Lung adenocarcinoma PDX (34, 1)	RNA-seq and WES	Identification of a subpopulation of *KRAS*+/low risk cells that were drug resistant	[45]
	LC2/ad and LC2/ad-R lung cancer cell lines (336, 7)	RNA-seq	Increased plasticity in gene expression among cells is associated with vandetanib resistance	[46]
*Brain*				
	*EGFR* amplified glioblastomas (50-60, 2)	CNV	Patterns of *EGFR* mutations differ among cells; heterogeneity may contribute to therapy resistance	[48]
	Glioblastomas (430, 5)^a^	RNA-seq	Variable *EGFR* CNVs and cells reflecting different subtypes are present in primary glioblastomas	[38]
*Colon*				
	Colon tumor and normal adjacent cells (63, 1)	SNV	Different mutational profiles found in two sub-clonal populations of cells may suggest bi-clonal origins	[49]
	HCT116 cell line (96, 1)	RNA-seq	SCS reveals cryptic mutations not detected in bulk tumor	[50]
*Bladder*				
	Muscle-invasive bladder transitional-cell carcinoma (66, 1)	SNV	Cell-lineage-specific mutations may initiate carcinogenesis and drive cancer progression	[51]
	Squamous cell carcinoma of the bladder (75, 1)	RNA-seq	Cell-to-cell heterogeneity in the expression of genes within cancer-related pathways may affect outcomes	[52]
*Kidney*				
	Clear cell renal cell carcinoma (20, 1)	SNV	ccRCC more genetically complex than predicted based on whole-tumor sequencing	[53]
	ccRCC primary carcinoma and paired metastasis propagated in PDX model (116, 1)	RNA-seq	Differential expression of targetable genes between cells supports multi-agent treatment strategy	[54]
*Blood*				
	Secondary AML (36, 3)	SNV	SCS identifies genomic complexity not seen in whole-tumor analysis and resolves clonal relationships	[55]
	Pediatric ALL (1479, 6)	SNV	CNVs precede somatic mutations; diversity of driver mutations affects clonal fitness	[56]
	B-cell ALL (276, 3)	CNV	CNVs not detected in bulk tumors are observed in single cells; CNVs develop in response to environmental stressors	[57]
	*JAK2*-negative myeloproliferative neoplasm (58, 1)	SNV	Lack of identifiable sub-clones suggests tumor is monoclonal, but large genetic distances exist between cells	[58]

*TNBC* triple negative breast cancer, *CNV* copy number variant, *ER* estrogen receptor, *HER2* human epidermal growth factor receptor 2, *SNV* single nucleotide variant, *RNA*-*seq* RNA sequencing, *PDX* patient-derived xenograft, *WES* whole-exome sequencing *KRAS* Kirsten rat sarcoma viral oncogene homolog, *EGFR* epidermal growth factor receptor, *SCS* single-cell sequencing, *ccRCC* clear cell renal cell carcinoma, *AML* acute myeloid leukemia, *ALL* acute lymphoblastic leukemia, *JAK2* Janus kinase 2

^a^These studies investigated transcriptomic differences in breast and glioblastoma stem cells isolated as single cells from the primary carcinomas

NGS technology is being used extensively to identify genetic variability associated with acquired resistance to chemotherapy, which has become a major barrier to successful cancer treatment. Large-scale RNA-seq on single cells from breast cancer cell lines has shown that cells exhibiting high variability in RNA transcripts, which was also evident at the protein level, possess increased metastatic capacity and survival following chemotherapeutic treatment [42]. Whole-transcriptome sequencing detected high heterogeneity in gene expression among individual cells from the MDA-MB-231 metastatic breast cancer cell line following exposure to paclitaxel (100 nM) for five days. Although most cells were killed, a small number of drug-tolerant cells survived, which expressed unique RNA variants influencing cell adhesion, cell surface signaling, and microtubule organization/stabilization [43]. These studies demonstrate that molecular heterogeneity at the single-cell level may have a significant impact on patient outcomes and that quantification of this heterogeneity will be vitally important to successful cancer treatment.

#### Adenocarcinoma of the lung

Adenocarcinoma of the lung is the most common histologic subtype of lung cancer, accounting for more than 40% of lung cancer incidence. Several studies have performed single-cell RNA-seq on lung cancer patients to investigate molecular heterogeneity at the single-cell level. Min et al. [44] examined 34 single cells from a lung adenocarcinoma PDX model, and after filtering out differentially expressed genes associated with xenografting and cell culture, identified a set of 64 genes associated with poor prognosis that stratified the adenocarcinoma cells into two groups. In a separate study, single lung adenocarcinoma cells from this same PDX were evaluated by RNA-seq and expressed mutation profiling to study how heterogeneous cell populations respond to anti-cancer treatments [45]. Combining the status of the Kirsten rat sarcoma viral oncogene homolog (*KRAS*) G12D (35G>A) mutation with the expression profiles of 69 genes associated with clinical prognosis classified the adenocarcinoma cells into four groups with different gene expression patterns. One group of cells that appeared cell-cycle quiescent and exhibited upregulation of ion channel transport genes survived exposure to chemotherapeutic agents and thus may be responsible for treatment failure. This study suggests that the actual cells responsible for drug resistance may be masked when analyzing large sections of the primary carcinoma, but single-cell RNA-seq data may be useful for detecting rare potentially drug-resistant sub-clones. Suzuki and colleagues conducted single-cell RNA-seq on 336 cells from seven lung adenocarcinoma cell lines to investigate how cellular heterogeneity influences drug response [46]. Focusing on the LC2/ad cell line and a derivative cell line (LC2/ad-R), which has acquired resistance to the multi-tyrosine kinase inhibitor drug vandetanib, showed that average gene expression levels changed more in LC2/ad-R cells than in LC2/ad cells in response to vandetanib treatment, potentially reflecting an acquired plasticity in the ability to respond to vandetanib. As seen in other single-cell studies, the great diversity in gene expression at the single-cell level, which may serve as a reservoir for cells to acquire drug resistance, cannot be detected with bulk tissue sequencing.

#### Glioblastoma

Glioblastoma multiforme is the most common brain and central nervous system malignancy, characterized by a poor prognosis with exceptionally low overall survival. Glioblastomas are biologically aggressive carcinomas that present unique clinical challenges due to rapid growth rates with widespread invasion throughout the brain and inherent resistance to traditional as well as targeted therapies [47]. Extensive cellular and molecular heterogeneity is a common feature of glioblastomas, including multiple alterations in the epidermal growth factor receptor (*EGFR*) gene that may affect treatment response. To characterize genomic heterogeneity in *EGFR*-amplified glioblastomas, Francis et al. conducted single-nucleus WGS on two glioblastomas with focal amplification of *EGFR* [48]. *EGFR* copy number was observed to be highly variable between single cells due to varying levels of *EGFR* amplification (5–200 copies), EGFRvII truncation (deletion of exons 14–15), and EGFRvIII deletion (deletion of exons 2–7). These data suggest that heterogeneity in the expression of oncogenic *EGFR* mutations may contribute to therapy resistance and combining multiple EGFR inhibitors that act through different mechanisms may be required in glioblastoma patients who carry multiple *EGFR* variants.

Patel and colleagues used single-cell RNA-seq on 430 cells from five primary glioblastoma neoplasms to systematically interrogate intratumor heterogeneity [38]. In agreement with the study described above by Francis et al. [48], several oncogenic variants of *EGFR* were detected within a single glioblastoma. Based on patterns of gene expression, all five tumors were found to consist of heterogeneous mixtures of individual cells corresponding to different glioblastoma subtypes defined by The Cancer Genome Atlas. Importantly, cell-to-cell variability was also detected in the expression of various signaling molecules and cell-surface receptors comprising pathways that may contribute to targeted-therapy resistance in glioblastoma. As higher levels of cell-to-cell subtype heterogeneity were associated with decreased patient survival, previously unrecognized heterogeneity may be an important factor contributing to the high mortality rates associated with glioblastoma.

#### Colon cancer

Unlike many types of human cancer, linear models of evolution have been developed for colon cancer, with mutations in genes such as adenomatous polyposis coli (*APC*) and tumor protein p53 (*TP53*) playing critical roles in tumor progression. WES performed on 63 single colon adenocarcinoma cells revealed two groups of tumor cells with distinct genetic profiles [49]. The major subgroup of tumor cells was characterized by a high frequency of *APC* and *TP53* mutations while in the minor subgroup, mutations in the cell division cycle 27 (*CDC27*) and polyadenylate-binding protein, cytoplasmic, 1 (*PABPC1*) genes were predominant. The authors concluded that this tumor was bi-clonal in origin, with each subpopulation deriving from separate ancestors; however, this conclusion has been questioned as not all cells in the major population had mutations in *APC* and *TP53* and mutations in *CDC27* and *PABPC1* were present in both groups, suggesting possible technical difficulties associated with WGA [16]. In a separate study, RNA-seq data generated on 96 single cells from the HCT116 colon cancer cell line were used to assess patterns of gene expression and detect enrichment of DNA variants in colon cancer-related pathways [50]. SNV data from the single isolated cells were mostly consistent with results obtained when the cell line was sequenced *en masse*, but single cells displayed an array of variants that were masked when many cells from the cell line were sequenced together (bulk sequencing). This study showed that single-cell RNA-seq of colon cancers may reveal cryptic genetic alterations in cancer-related genes, enrichment of certain functional pathways, and presence of fusion proteins that may play important roles in the development of colon cancer.

#### Urinary system cancers

Bladder cancer accounts for nearly 5% of all new cancer cases in the United States and is responsible for approximately 3% of all cancer deaths. Bladder cancer is marked by heterogeneity in the types of carcinomas observed in patients and the presence of infiltrating normal cells. Single-cell exome sequencing of 66 individual tumor cells from a muscle-invasive bladder transitional-cell carcinoma revealed that all cells were descended from a common ancestral cell, but subsequent genomic evolution created variability that could partition the cells into two distinct groups [51]. The authors hypothesized that the bladder cancer cells were subjected to selective pressure and accumulated mutually-exclusive driver mutations within these cell lineages during development. The projected timing of key mutations during cancer growth suggests that mutations in cancer-associated genes may initiate carcinogenesis and lead to genetically-distinct cell lineages that influence resistance to treatment.

To evaluate cellular heterogeneity in gene expression within a squamous cell carcinoma of the urinary bladder, Zhang et al. subjected 75 individual cancer cells to RNA-seq [52]. Cell-to-cell heterogeneity was detected for multiple genes in important cancer-related pathways, including the mitogen-activated protein kinase (*MAPK*), Janus kinase/signal transducers and activators of transcription (JAK-STAT), Notch, phosphoinositide 3-kinase (*PI3K*), and vascular endothelial growth factor (*VEGF*) pathways. Because these pathways represent important targets for anti-cancer therapeutics, heterogeneity in expression may affect tumor response to therapy and patient survival.

Renal cell carcinoma accounts for more than 200,000 new cancer cases and over 100,000 deaths worldwide each year. Clear cell renal cell carcinoma (ccRCC), the most common form of renal cell carcinoma, is characterized by a relatively low mutation rate with few mutations shared among patients. To investigate intratumor heterogeneity at the individual cell level in ccRCC, WES was conducted on 20 single ccRCC cells from a 59-year-old male patient [53]. Phylogenetic analysis suggested that progression from normal to cancer cells occurred quickly. Although no significant sub-clonal populations of cells were detected within the tumor, there were many rare mutations, each present in only a few cancer cells. These mutations would not have been detected using whole-tumor sequencing. This study provided an important view of the intratumor genetic landscape of a ccRCC carcinoma at the single-cell level and revealed that renal carcinomas may be more genetically complex than previously thought.

To examine transcriptional heterogeneity during metastatic progression and the activation of signaling pathways influencing drug responsiveness, single-cell RNA-seq was performed on a primary ccRCC carcinoma and a paired lung metastasis following propagation in a PDX model [54]. This patient was not responsive to sequential therapies, including pazopanib, everolimus, and high-dose interleukin-2. The RNA-seq results revealed significant variability in expression and activation of pathways targeted by therapy, such as the EGFR and c-Src proto-oncogene pathways, between the primary carcinoma and the metastasis, and among individual cancer cells within both tumors. Heterogeneity in the activation status of the EGFR and Src pathways corresponded to variability in drug sensitivity at the individual cell level. High-resolution transcription profiling of single cells established the molecular basis for treatment resistance and led the authors to propose that combination therapy with afatinib and dasatinib may be a more effective treatment option than monotherapy for metastatic renal cell carcinoma.

#### Hematopoietic tumors

Hematopoietic and lymphoid tissue malignancies affect the blood, bone marrow, and lymphatic system. To further examine genomic complexity in hematopoietic cancers previously studied by WGS of bulk tumor samples, Hughes and colleagues performed targeted sequencing to genotype more than 1900 SNVs in single cancer cells from three patients initially diagnosed with myelodysplastic syndrome who progressed to secondary acute myeloid leukemia, the most common form of acute leukemia in adults [55]. SCS identified genomic complexity not evident in the whole-tumor analysis and improved the ability to resolve clonal relationships compared to sequence generated from unfractionated tumor samples. To delineate the clonal structure and evolutionary history of acute lymphoblastic leukemia (ALL), targeted sequencing of a panel of SNVs, deletions, and immunoglobulin heavy chain sequences was performed on 1479 single cells from six children with pediatric ALL [56]. As seen with other types of cancer, ALL carcinomas were characterized by distinct clonal populations of cells where alterations in copy number preceded the occurrence of SNVs. Phylogenetic analysis revealed that *KRAS*-associated driver mutations occurred late in tumor development and facilitated the expansion of certain clones, which became dominant but did not completely outcompete all of the other clones in each patient. Separately, Bakker et al. used single-cell WGS to examine karyotype dynamics in three children with chromosomally-unstable B cell ALL [57]. Traditional cytogenetics conducted at the time of diagnosis characterized the ALL carcinomas as displaying different levels (low, intermediate, and high) of aneuploidy. SCS identified subpopulations of cells within each tumor that harbored copy number alterations not detected in whole-tumor analysis. When cells from the ALL tumor with intermediate levels of aneuploidy were engrafted into immunodeficient mice, changes in copy number were observed, suggesting that copy number heterogeneity in individual cells may evolve in response to stressors, such as a new microenvironment or exposure to therapy.

Essential thrombocythemia (ET) is one of several myeloproliferative neoplasms in which sustained proliferation of megakaryocytes leads to an excess of circulating thrombocytes (platelets). Although more than half of all ET patients carry mutations in the Janus kinase 2 (*JAK2*) gene, mutations in other genes are known to affect disease phenotype and clinical outcome. WES of 58 single cancer cells from a *JAK2*-negative ET patient was used to examine clonal composition of the neoplasm and identify genes involved in disease progression [58]. The authors identified 18 genes hypothesized to play a role in tumor development and concluded that the disease was monoclonal in origin. However, these conclusions were contradicted by phylogenetic analyses, which showed large genetic distances between cells, and therefore it is unclear if these differences reflect real genomic diversity or technical artifact.

SCS has been useful for revealing molecular heterogeneity among individual cells of primary carcinomas from a variety of human cancers that would not be detectable with bulk tumor sequencing. At the single-cell level, most primary tumors are polyclonal due to punctuated clonal evolution where copy number alterations serve as founder mutations and additional CNVs and/or point mutations occur later in tumor development. These subsequent mutations are restricted to subpopulations of cells where they contribute to clonal fitness and thus influence resistance to treatment and patient survival.

### Circulating and disseminated tumor cells

A summary of SCS studies on CTCs and DTCs from a variety of human cancers is presented in Table 4. Substantial evidence suggests that distinct subpopulations of stem-like cells mediate many aspects of cancer biology, including metastasis and therapeutic resistance [59]. CTCs are viable cells that are shed from a primary carcinoma and circulate throughout the bloodstream, carrying genetic alterations found in the primary tumor [60]. The presence and/or abundance of CTCs in whole blood has been shown to be an independent predictor of poor survival and an unfavorable response to treatment in numerous cancer types [61], and the persistence of disseminated cells in bone marrow after adjuvant therapy is significantly associated with increased risk for recurrence and mortality [62].

**Table 4:** Summary of single-cell sequencing studies of CTCs and DTCs

Cell type	Tumor type (number of cells, patients)	Data type	Results	Reference
*CTCs*				
	Colorectal (37, 6)	Targeted sequencing	Most mutations in CTCs are present in sub-clonal populations of the primary tumor or metastases, but some mutations are exclusive to CTCs	[67]
	Lung (68, 11)	WES/WGS	CNVs in CTCs are dissimilar between cancer subtypes; patterns of SNVs and INDELs in CTCs change during treatment, but CNVs remain constant	[68]
	Prostate (99, 1)	WGS	SNVs and structural variations in CTCs are also present in primary tumors or metastases	[69]
	Prostate (25, 2)	WES	The majority of mutations in CTCs are also present in the primary tumor and metastases	[70]
	Breast (14, 4)	Targeted sequencing	High levels of heterogeneity in CTCs within and between patients as well as before and after treatment	[71]
	Breast (115, 18)	Targeted sequencing	In some patients heterogeneity of *PIK3CA* mutations is observed among CTCs and between CTCs and the primary tumor	[72]
	Breast (11, 2)	Targeted sequencing	Some CTCs carry the same *TP53* mutation(s) as the primary carcinoma, other CTCs carry different mutations	[73]
	Breast (185, 12)	Targeted sequencing	CTCs show genetic heterogeneity of *PIK3CA* mutations over time and discordance between DTCs and metastases	[74]
	Breast (22, 2)	RNA-seq	HER2 + CTCs may arise in HER2- breast cancer patients and may contribute to progression and drug resistance	[75]
	Prostate (77, 13)	RNA-seq	Heterogeneity in expression of androgen receptor mutations between CTCs within patients may influence treatment response	[77]
*DTCs*				
	Breast (24, 1)	Targeted sequencing	DTCs show genetic discordance of *PIK3CA* mutations versus CTCs and metastases; mutations are stable during cell culture	[74]
	Breast (2, 2)	WGS	In one patient, DTC was highly concordant with the non-complex primary tumor; DTC from complex primary tumor showed greater genetic divergence	[82]
	Neuroblastoma (144, 10)	Targeted sequencing	Mutational status for the *ALK* gene is concordant between the primary tumor and DTCs for all patients	[84]
	Breast (63, 6)	WGS	Some DTCs originate from clones in the primary carcinoma, other DTCs arise from LN metastases	[85]

*CTC* circulating tumor cell, *WES* whole-exome sequencing, *WGS* whole-genome sequencing, *CNV* copy number variant, *SNV* single nucleotide variant, *INDEL* insertion/deletion polymorphism, *PIK3CA* phosphatidylinositol-4,5-bisphosphate 3-kinase, catalytic subunit alpha, *TP53* tumor protein p53, *DTC* disseminated tumor cell, *RNA*-*seq* RNA sequencing, *HER2* human epidermal growth factor receptor 2, *ALK* anaplastic lymphoma kinase, *LN* lymph node

Only certain CTCs are believed to be capable of forming successful metastases. Recent evidence suggests that some CTCs, referred to as circulating cancer stem cells, exhibit a stem-cell-like phenotype and may possess metastasis-initiating capabilities associated with resistance to therapy [63, 64]. Because CTCs that display stem cell characteristics may initiate successful metastases, it is important to characterize these cells, which are easily accessible in peripheral blood, for their usefulness in predicting cancer progression, metastasis, and treatment response.

#### Circulating tumor cells

SCS is a useful technique for improving our understanding of clonal evolution in human cancers, as well as molecular changes that occur in disseminated cancer cells, which may drive metastasis and lead to development of therapeutic resistance. Numerous studies have shown that mutational profiles identified by NGS may be similar in primary carcinomas, metastases, and CTCs in patients with a variety of cancer types, but important molecular heterogeneity has been detected, suggesting potential utility of CTCs in patient care (reviewed in [65, 66]).

NGS of 68 cancer-associated genes in individual CTCs from patients with stage IV colorectal cancer found that most mutations, particularly those in driver genes, observed in the primary tumor and metastatic deposits were also present in CTCs, suggesting that the mutational spectrum of complex tumor genomes can be inferred from CTCs [67]. Similarly, WES of single CTCs in lung cancer patients detected reproducible CNVs that were similar to those in metastatic deposits of the same patient [68]. In patients with prostate cancer, 70% (51/73) to 86% (197/229) of all mutations observed in individual CTCs were also found in the primary tumor and metastasis [69, 70].

SCS has been used to identify within-patient genomic heterogeneity among single CTCs isolated from blood of breast cancer patients. For example, mutational heterogeneity in the *TP53* gene, platelet-derived growth factor receptor, alpha (*PDGFRA*), phosphatidylinositol-4,5-bisphosphate 3-kinase, catalytic subunit alpha (*PIK3CA*), and other genes has been observed among individual CTCs from women with metastatic breast cancer [71, 72]. Similarly, the mutational status of *TP53* has been shown to vary among CTCs in breast cancer patients, with some CTCs carrying the same mutation(s) as the corresponding primary carcinoma, while other CTCs carry different mutations [73].

Mutational heterogeneity present in a primary carcinoma is often reflected in the genomes of CTCs; however, further genomic changes that promote successful metastasis may occur exclusively in CTCs and DTCs [74]. Such heterogeneity at the single-cell level likely reflects dynamic and ongoing mutational changes that occur during disease progression in a constantly evolving cancer genome. Therefore, the genomic signatures of many individual CTCs from a cancer patient may be more informative than traditional biopsies of the primary tumor for designing targeted therapies and monitoring therapeutic response.

Optimal therapeutic strategies in breast cancer patients are highly dependent on the behavior and resilience of CTCs, which may be influenced by patterns of gene expression. Similar to genomic heterogeneity, cell-to-cell variability in patterns of gene expression has been identified among individual CTCs. In women initially diagnosed with human epidermal growth factor receptor 2 (*HER2*)-negative breast cancer, RNA-seq of individual CTCs documented the emergence of HER2 + CTCs [75]. The persistence of discrete populations of HER2+ and HER2 − CTCs, which have the capacity to interconvert spontaneously, may contribute to progression of breast cancer and acquisition of drug resistance. Similarly, single-cell transcriptome analysis of CTCs revealed heterogeneity in the expression of genes associated with metastasis and induction of the EMT, where epithelial cells transition to a more mesenchymal phenotype, which increases invasiveness and resistance to apoptosis [76].

Men with prostate cancer may be initially responsive to androgen receptor (*AR*) inhibitors, but in some patients, single-cell RNA-seq of individual CTCs detected heterogeneity in the expression of *AR* gene mutations and activation of non-canonical (β-catenin-independent) Wnt signaling, which may promote invasiveness and malignant progression, thereby contributing to treatment failure [77]. In pancreatic ductal adenocarcinoma, RNA-seq has been used to compare genome-wide expression profiles of single cells disaggregated from the primary carcinoma with corresponding CTCs in a mouse model of pancreatic cancer [78]. Compared with cells from the primary tumor, CTCs showed enrichment of some genes associated with stem cells and reduced expression of epithelial markers (E-cadherin and Mucin 1). Within CTCs, a high degree of heterogeneity was evident in the expression of mesenchymal transcripts, platelet-derived markers, and proliferative gene signatures.

#### Disseminated tumor cells

Research on the role of DTCs in bone marrow of cancer patients has increased in recent years because the dissemination of cells from a primary carcinoma is believed to be a critical step in the process of disease progression and formation of distant metastases. The presence of single DTCs in bone marrow has been established as a strong predictor of distant disease-free survival and breast cancer-specific survival in breast cancer patients [79]. Patients with non-metastatic breast cancer remain at significant risk of relapse, even after complete surgical excision of the primary carcinoma, likely due to the persistence of disseminated cancer cells [80].

Disseminated cancer cells detected in bone marrow of patients with breast cancer have been found to express proteins characteristic of cancer stem cells [81]. DTCs are similar to CTCs in that they arise from sub-clonal populations of cells in the primary carcinoma and undergo further molecular changes after dissemination [82]. Cancer biomarkers and genetic variation in both CTCs and DTCs may evolve during disease progression, and significant molecular discordance with important therapeutic implications may develop between the primary tumor and disseminated cells [83].

SCS studies of DTCs have been limited, presumably because of the invasive surgical procedures needed to collect these cells. In one study, Carpenter and colleagues isolated 144 disseminated cells from bone marrow of patients affected with neuroblastoma [84]. In patients carrying a mutation in the anaplastic lymphoma kinase (*ALK*) gene in their primary tumor, single-cell WGA and sequencing detected the same mutation in single DTCs from bone marrow. Demeulemeester and colleagues used SCS to trace the origin of 63 single disseminated cells from six non-metastatic breast cancer patients [85]. Approximately one-half of the DTCs which morphologically resembled cancer cells were found to be disseminated from the primary tumor; however, some of the remaining cells displayed normal copy-number profiles, while other cells had CNVs that were genetically different from the primary tumor. Reconstructing evolutionary relationships between the primary tumor and DTC genomes showed that some DTCs originated from the predominant clone in the primary carcinoma, other DTCs arose from less prevalent lineages in the primary tumor, and a few DTCs descended from minor clones observed in the axillary lymph node metastases.

## Single-cell sequencing in clinical practice

Targeted therapeutics are designed to focus on actionable mutations detected in a biopsy of the primary tumor, but these “actionable” mutations may no longer drive disease progression once tumor cells disseminate from the primary carcinoma and undergo unique genomic changes. The ability of single-cell sequencing to delineate the genomics and transcriptomics of circulating and disseminated cancer cells holds great promise for making meaningful improvements in personalized oncology over the next several years. To date, SCS has been used primarily in the research setting; however, there may be a number of clinical applications, including diagnosis, prognosis, treatment decisions, and monitoring [86]. An intriguing use of SCS would be early disease diagnosis through the analysis of bodily fluids such as blood or urine. Through regular noninvasive monitoring of high-risk patients, single disseminated cancer cells may be detectable at an early stage of disease before a cancerous lesion could be visualized with current imaging technologies. Identification of clinically-actionable mutations at an early stage could lead to targeted treatment before tumor heterogeneity and multiple genomically-distinct clones that are resistant to therapy can evolve. Additionally, improvements in SCS technologies will enable analyses of small tumors which previously were too small to analyze using bulk sequencing approaches. As demonstrated by SCS of primary carcinomas, single biopsies may fail to adequately account for intratumor heterogeneity. Assessing genomic heterogeneity within the primary tumor or among disseminated cells would allow for the calculation of diversity scores which may be used prognostically, with higher intratumor heterogeneity associated with less favorable outcomes [65, 87].

SCS may also be used to optimize treatment. The ability to identify common mutations throughout a carcinoma could permit use of single agents that target the bulk of the tumor, while assaying heterogeneous actionable mutations could lead to implementing combinatorial approaches that target sub-clonal populations of cells [86]. For cancer treatment, the most promising clinical use of SCS is the analysis of CTCs, which may provide a non-invasive method for clinicians to monitor response to therapy before tumors become symptomatic or detectable through traditional approaches. Serial analysis of individual CTCs isolated from blood samples taken over the course of treatment may be used to identify new mutations that emerge in response to therapy which influence disease progression or therapeutic resistance [88], enabling oncologists to alter treatment accordingly. Targeted elimination of circulating tumor cells with stem-cell-like expression profiles could prevent the colonization of secondary sites and formation of metastases.

Despite the potential utility of SCS in clinical cancer care, several current limitations need to be addressed before SCS can be used routinely in clinical practice. In the clinical environment, cancerous tissues excised from the body have traditionally been prepared for pathological examination by fixing the tissue in formalin and embedding in paraffin. However, most single-cell isolation and sequencing methods have been designed for use with suspensions of live cells acquired from fresh tissues [86]. Although the nuclear membrane is resistant to freezing and thawing, allowing individual nuclei to be isolated from nuclear suspensions derived from frozen tissues for DNA sequencing [89], fresh tissue is currently needed for single-cell RNA-seq. To implement SCS in the clinic, new tissue collection and handling protocols will have to be established and validated at medical centers and treatment facilities. Single-cell WGA and WTA techniques currently being used in the research setting have technological limitations, and an important challenge to implementing SCS in the clinic is overcoming errors that may be introduced by amplifying the minute amount of DNA or RNA in a single cell and properly validating the sequencing results. Improved technologies as well as new computational methods will be needed before SCS can reliably distinguish technical errors from true biological variability and generate valid results for informing patient care [7, 90].

Currently, the cost of SCS prohibits large-scale implementation in the clinical setting, particularly because added costs for computational analysis will be incurred and assessment of numerous individual cells is often necessary. Hundreds of single cells may need to be sequenced, depending on a variety of factors, including the state of disease progression, tumor heterogeneity, and rarity of clinically important clones. Few insurance companies provide coverage for SCS, particularly for cancer patients, and until the clinical validity and clinical utility of SCS are unequivocally demonstrated, patients will have to pay out of pocket for these services. Large studies assessing clinical validity and robust decision models regarding patient outcomes are needed to influence payer coverage decisions regarding SCS [91].

A major obstacle complicating the introduction of SCS into the clinical environment is the lack of onsite oncologists or physicians who sufficiently understand the sequencing results and are able to translate those results into clinical action. Questions being asked by clinicians include: (1) how to interpret and apply SCS results to individual patients, (2) how to translate DNA or RNA variation within single cells into definable clinical phenotypes, and (3) how to use SCS results to predict patient response to treatment [92]. Despite the growing availability of clinically-useful DNA- and RNA-based tests, ethical issues of sharing with the patient secondary (incidental) findings—genetic alterations associated with conditions or diseases unrelated to the patient’s present condition—remain unresolved [93]. In addition, although the cost of SCS continues to decrease, the time required for completing the isolation of single cells, DNA amplification, NGS, and data interpretation remains a significant obstacle. One recent study examining the integration of WGS analysis into cancer care found that results were clinically actionable in ~55 days, considerably longer than the 10- to 14-day time frame that most patients and physicians would find acceptable for diseases such as cancer where rapid treatment decisions are highly desirable [94].

The Individualized Molecular Pancreatic cancer Therapy (IMPaCT) trial, designed to improve outcomes using genomic information to guide treatment decisions for patients with advanced pancreatic cancer, found that a complex infrastructure and multidisciplinary team consisting of a genetic pathologist, oncologist, genetic counselor, research coordinator, and project manager were necessary to collect and process biospecimens, conduct genomic analyses, and return results in a clinically relevant timeframe [95]. The median time from consent to return of validated results was 21.5 days (range 7–82 days). The trial concluded that current barriers to implementing NGS technology in the clinic are surmountable with the appropriate personnel and sufficient resources.

## Conclusions

Over the next several years, advancements in the isolation of single viable cells, as well as WGA, NGS, and computation methods will be needed to improve the clinical utility of SCS [4]. The ability to amplify and sequence RNA molecules other than polyadenylated mRNAs, such as long non-coding RNAs and micro RNAs, will provide valuable information on gene regulation. New methods to simultaneously amplify and sequence genomic DNA and full-length mRNA from the same cell may provide powerful tools for assessing the effects of genomic variation on gene expression profiles [96, 97]. Likewise, the ability to couple genome-wide methylation [98] and/or proteomic [99] analysis with single-cell DNA- and RNA-sequencing from individual cells may reveal mechanisms by which genetic and epigenetic modifications regulate transcriptional heterogeneity in cancer. Fluidic systems to simultaneously isolate and analyze millions of cells in parallel may provide a comprehensive view of cancer development and response to therapy within each patient. Finally, localizing the spatial organization of gene and protein expression within a single cell may be key to determining the behavior and survival of individual cancer cells during therapy [100].

SCS is providing new insight into the biological and molecular complexity of cancer, yet despite major recent advancements, the extent of genomic and transcriptomic heterogeneity at the individual cell level in human cancer remains largely uncharacterized. Heterogeneity in cancer patients is known to be dynamic and to evolve unpredictably during disease progression, which creates a significant challenge for modern cancer treatments. SCS has the potential to create a paradigm shift in cancer care to precision (personalized) treatment where heterogeneity is thoroughly characterized prior to and during treatment. Cancer immunotherapy, in particular, may benefit from single-cell methods that define the role of innate heterogeneity in the development of immune resistance and monitor the response of individual cancer cells to immune-regulatory agents. Integrated SCS approaches may provide important new insights into cancer evolution and unveil new avenues for dissecting the complex activation of signaling pathways that cause heterogeneous cellular responses during treatment.

## Abbreviations

*ALK*: anaplastic lymphoma kinase
ALL: acute lymphoblastic leukemia
APC: adenomatous polyposis coli
*AR*: androgen receptor
ccRCC: clear cell renal cell carcinoma
CD45−: leukocyte common antigen 45−
*CDC27*: cell division cycle 27
cDNA: complimentary DNA
CEL-seq: cell expression by linear amplification and sequencing
CNV: copy number variant
CTC: circulating tumor cell
DOP-PCR: degenerate oligonucleotide-primed polymerase chain reaction
DTC: disseminated tumor cell
*EGFR*: epidermal growth factor receptor
EMT: epithelial-to-mesenchymal transition
EpCAM+: epithelial cell adhesion molecule
ER: estrogen receptor
ET: essential thrombocythemia
*HER2*: human epidermal growth factor receptor 2
*JAK2*: Janus kinase 2
JAK-STAT: Janus kinase/signal transducers and activators of transcription
*KRAS*: Kirsten rat sarcoma viral oncogene homolog
LCM: laser capture microdissection
MALBAC: multiple annealing and looping based amplification cycles
*MAPK*: mitogen-activated protein kinase
MDA: multiple-displacement amplification
mRNA: messenger RNA
NGS: next-generation sequencing
*PABPC1*: polyadenylate-binding protein, cytoplasmic, 1
*PDGFRA*: platelet-derived growth factor receptor, alpha
PDX: patient-derived xenograft
*PI3K*: phosphoinositide 3-kinase
*PIK3CA*: phosphatidylinositol-4,5-bisphosphate 3-kinase, catalytic subunit alpha
RNA-seq: RNA sequencing
SCS: single-cell sequencing
SMART-seq: switching mechanism at the 5′-end of the RNA transcript
SNV: single nucleotide variant
STRT: single-cell tagged reverse transcription
*TP53*: tumor protein p53
UMI: unique molecular identifier
*VEGF*: vascular endothelial growth factor
WES: whole-exome sequencing
WGA: whole-genome amplification
WGS: whole-genome sequencing
WTA: whole-transcriptome amplification
